# Invertebrate biodiversity in cold groundwater fissures in Iceland

**DOI:** 10.1002/ece3.5213

**Published:** 2019-05-03

**Authors:** Jónína H. Ólafsdóttir, Jóhann G. Þorbjörnsson, Bjarni K. Kristjánsson, Jón S. Ólafsson

**Affiliations:** ^1^ Hólar University College Sauðárkrókur Iceland; ^2^ Marine and Freshwater Research Institute of Iceland Reykjavík Iceland

**Keywords:** biofilm, macrozoobenthos, species assemblages, spring, substrate

## Abstract

Iceland has an abundance of fissures that are parallel to the Mid‐Atlantic Ridge where bedrock cracks as a result of continental rifting. Some fissures penetrate the aquifer and expose the groundwater within the bedrock, becoming springs. As such, groundwater fissures have uniform and constant physical and chemical environment but they can differ greatly in morphology. In addition, there is often great variation in depth within fissures and substrate types contrast between vertical rock wall and more heterogenous horizontal bottom. The variation in morphological environment may create dissimilar habitats with unique characteristics and/or influence distribution of resources. Our objective was to study macrozoobenthos communities in cold groundwater fissures in Iceland in relation to physical habitat by comparing invertebrate diversity and density both between fissures with different morphological characteristics as well as between substrate types and depths within fissures. Samples were collected in two fissures in SW Iceland, Silfra and Flosagjá. Assemblages were similar between fissures except for higher densities of cladocerans in Flosagjá fissure. Within fissures, there was significant difference in Shannon diversity between substrate types in Flosagjá, and ostracods were found in significantly higher densities on the bottom. The distribution of all other taxa groups was homogenous in both fissures regardless of depth gradient and substrate. Invertebrates were found to be living within and around a biofilm that covered the entire substrate. These biofilm mats are made from Cyanobacteria and benthic diatoms, which are successful under low light conditions and may minimize any effect of the heterogeneous habitat creating a uniform and suitable microhabitat for invertebrates regardless of depth and substrate type.

## 1 INTRODUCTION

In freshwater systems, invertebrates merit special consideration as secondary producers are important for many ecological mechanisms (Richardson & Jackson, 2002). Too often however, invertebrates are overlooked and their role in ecosystem functioning is not considered until sudden ecological changes have occurred (Covich, Palmer, & Crowl, 1999). In freshwater systems in Iceland, invertebrate diversity tends to be greatest in spring‐fed lakes and rivers compared to other freshwater systems (Gíslason, Ólafsson, & Aðalsteinsson, 1998; Malmquist et al., 2003). Biodiversity and density are generally high in springs due to environmental stability and complex habitat structure, which offers abundant and variable microhabitats for invertebrates (Cantonati, Gerecke, & Bertuzzi, 2006; Glazier, 1991). As a result, springs often contain a larger number of rare, endemic, and endangered species than other aquatic habitats (Cantonati, Füreder, Gerecke, Jüttner, & Cox, 2012). Despite this, the study of invertebrates in these systems is insufficient and springs remain the least studied of aquatic ecosystems (Ferrington, 1998). Springs are also typically the least protected which leaves them more vulnerable to anthropogenic impact (Cantonati et al., 2012).

**Figure 1:**
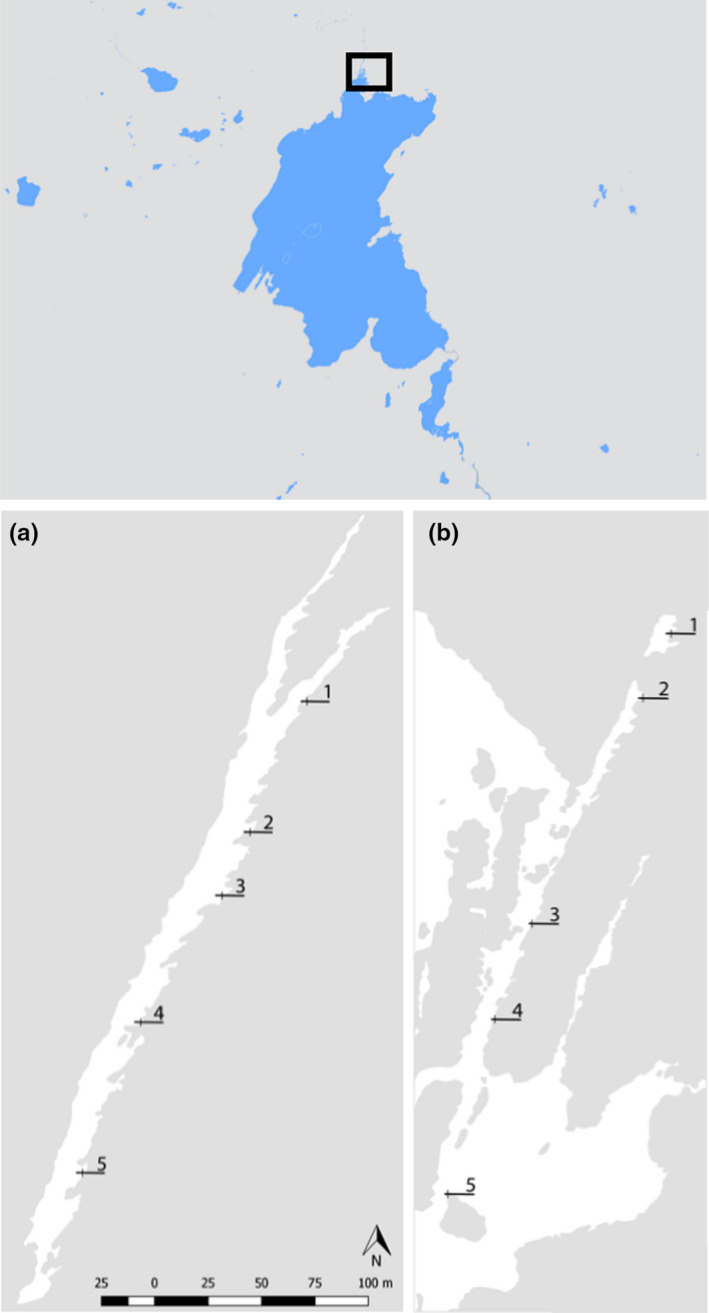
A map of Lake Þingvallavatn in SV‐Iceland and the two groundwater fissures studied, Flosagjá (a) and Silfra (b). The approximate location of the two fissures is defined by a black rectangle. The location and number of transects within fissures are specified with black lines

Most of the species found in springs are macrozoobenthos, and in cold groundwater systems in Iceland, chironomid larvae are the dominant taxon (Govoni, Kristjánsson, & Ólafsson, 2018). Springs are most common within the neovolcanic rock formations, originating from a rifting tectonic plate boundary and mantle plume, and these geological processes have caused the formation of numerous fissures (Jóhannesson & Sæmundsson, 2009; Marshak, 2008). Some of Iceland's rift fissures penetrate the aquifer and provide an opening into groundwater and thus act as spring habitats. Most of the fissures have cold and clear water (Ólafsson, 1992) and resemble freshwater systems in the High Arctic (Rautio, Bayly, Gibson, & Nyman, 2008). Since the fissures are often narrow and can reach up to 60 m in depth (Chowdury, 1998), most of the substrate is vertical rock wall while horizontal substrate is a smaller strip at the bottom (Figure 1). The bottom of fissures is often covered with rubble that has fallen from the walls. In some areas, larger pieces of rock form caverns with dim light conditions and/or caves where light does not penetrate. The fissures have been thoroughly studied in a geological and geophysical aspect (Guðmundsson, 1986), but the ecosystems of water‐filled fissures in Iceland are poorly known.

Our objective was to assess the biodiversity in cold groundwater fissures in Iceland in relation to their morphological habitat. Fissures in the same location typically have similar physical and chemical properties, and many environmental factors are uniform and constant as is characteristic of spring systems (Ólafsson, 1992; Van der Kamp, 1995). However fissures can differ greatly in morphology such as degree of cavern formation, uniformity of the bottom, and connectivity to other systems (some fissures are landlocked while others connect to water bodies). This variation may shape the diversity and density of invertebrates found within them.

Habitat heterogeneity has been shown to influence invertebrate community structure in springs (Ferrington, 1998). Within fissures, there is often great variation in depth and substrate types contrast between vertical rock wall and more heterogenous horizontal bottom. Light is absorbed and scattered in water which influences the distance it can travel (Jonasz & Fournier, 2007). Therefore, depth could influence light availability to varying degrees which in turn would affect primary production and available resources for benthic heterotrophs. Also, substrate types within fissures may create distinct niches and influence the distribution of organisms. Many studies on freshwater ecosystems have found that substrate is a principal environmental variable shaping macroinvertebrate assemblages (Alexander & Allan, 1984; Arunachalam, Nair, Vijverberg, Kortmulder, & Suriyanaraynan, 1991; Cobb, Galloway, & Flannagan, 1992; Duan, Wang, & Tian, 2008; Erman & Erman, 1984; Reice, 1980) although few of these studies have focused on continuous rock substrate. The different types of substrate found in fissure may influence the distribution of organisms and the availability of resources. For example, detritus is likely to accumulate readily on the bottom while little detritus would settle on rock walls. Since detritus is an important food source for most collectors (Hall & Meyer, 1998; Petersen, Gíslason, & Vought, 1995; Vannote, Minshall, Cummins, Sedell, & Cushing, 1998) its distribution can create distinct niches and may reflect the microhabitat of collectors (Covich et al., 1999).

As habitat diversity is known to influence biodiversity (Covich et al., 1999), we investigated whether the morphological habitat in fissures shapes assemblages of macroinvertebrates. We compared the density and diversity of invertebrate assemblages between fissures with different morphological characteristics. Furthermore, we investigated the distribution of invertebrates within fissures by comparing density and diversity between substrate types (rock wall and bottom) and at variable depths. Here we provide a detailed first look at macroinvertebrate communities within cold groundwater fissures in Iceland.

## 2 MATERIALS AND METHODS

**Figure 2:**
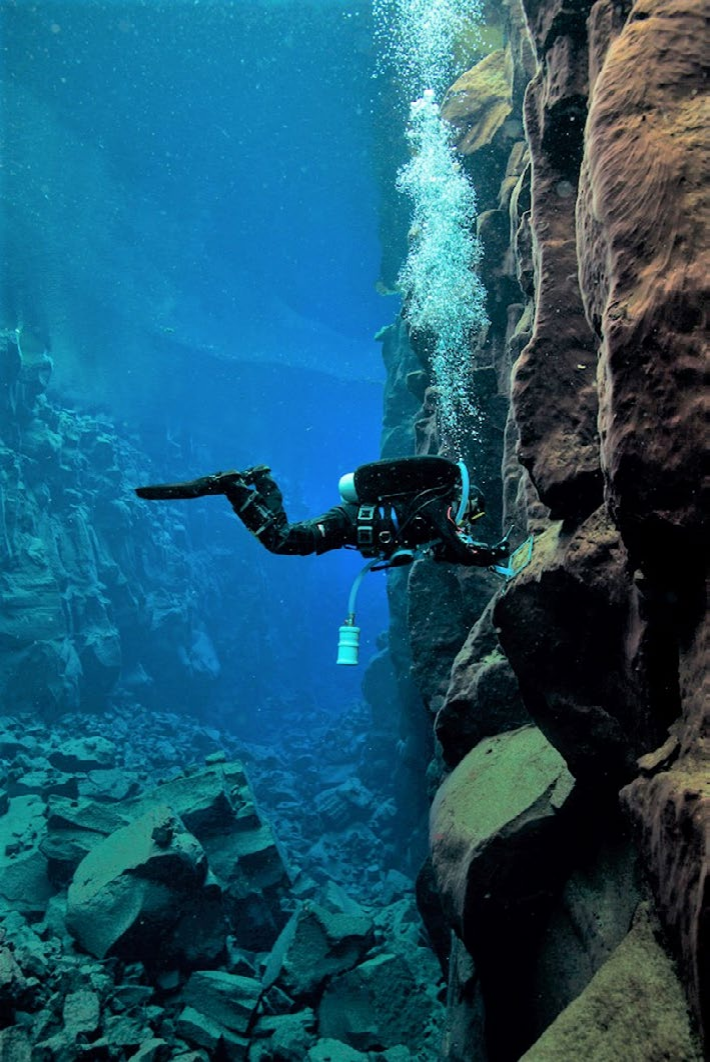
Photograph of a diver in Flosagjá fissure in Þingvellir National Park SW Iceland. Photograph by Gísli Arnar Guðmundsson

**Figure 3:**
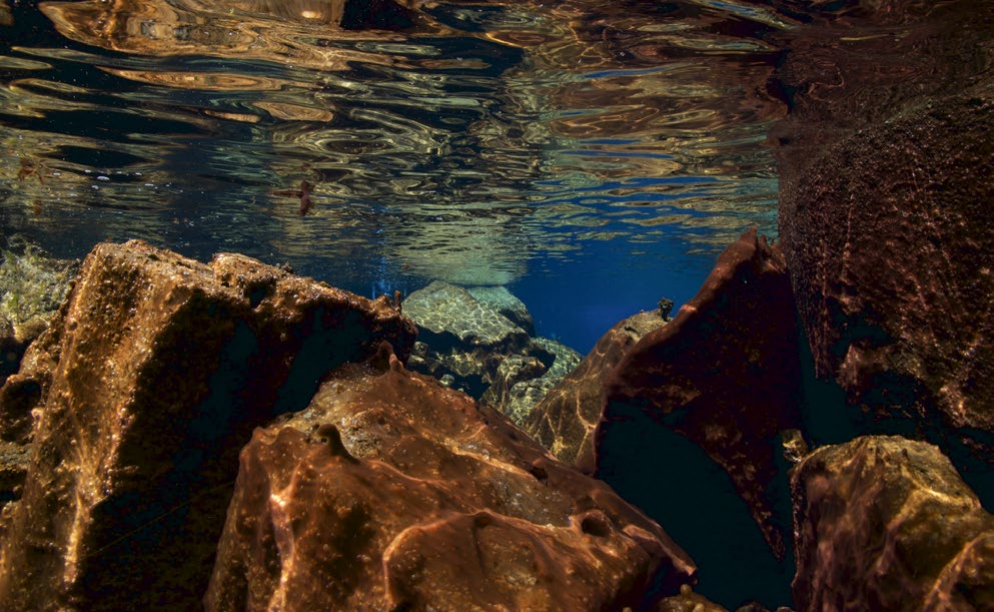
Benthic biofilm covers the substrate in fissures. Photograph by Gísli Arnar Guðmundsson from Flosagjá fissure

Macroinvertebrate samples were collected in June and July 2013 from two large fissures in Þingvellir National park in southwest Iceland (Figure 1). Flosagjá (64°15′46 N, 21°06′46 W) and Silfra (64°15′14 N, 21°07′05 W) are located in 9,000‐year‐old Eldborg lava flow as part of the Hengill fissure swarm (Sigurðsson & Sigbjarnason, 2011; Sinton, Grönvald, & Sæmundsson, 2005; Thors, 1990). The two fissures are located 300 meters apart. Flosagjá is landlocked and is among the largest fissures in the area. A 400 m section between two collapses was sampled, reaching a maximum depth of 18 m. Silfra fissure is one of the largest springs opening directly into Lake Þingvallavatn (Malmquist, 2012). Its open water section is about 300 m long with a maximum depth of roughly 20 m while several caverns and caves reach greater depths. Much of the substrate in the fissures is vertical rock wall (Figure 2) and covered in benthic biofilm (Figure 3). Samples were taken at five randomly selected depth transects within each fissure. On each transect, five replicate samples were collected on rock wall and bottom. Samples from rock wall were collected at 1m while the depth of bottom sampling stations was variable between 4–10 m as determined by the depth of the randomly selected transect. All samples were acquired by SCUBA diving. Invertebrates and surrounding biofilm were collected into sampling containers using a brush and pump sampler (Þorbjörnsson, Ólafsdóttir, & Kristjánsson, 2018) from within a 0.04 m^2^ frame taken randomly in the area of each sampling site (Figure 2). Sampling containers were collected into net bags and brought up to the surface where the material was filtered through a 125 µm sieve and preserved in 70% ethanol.

In the laboratory, invertebrates were counted and identified to various taxonomic groups. The four most common invertebrate groups (chironomids, cladocerans, copepods, and ostracods) were identified to the lowest taxonomic level possible. Only a subsample of copepods was identified to species level, and therefore, the subclass “Copepod” was treated as a taxonomic group in statistical analysis. Detailed species identification followed taxonomic keys by Cranston (1982) and Schmid (1993) for Chironomidae, Alonso () for Cladocera, Alekseev and Defaye (2011) for Copeoda, Meisch (2000) for Ostracoda, Gíslason (1979) for Trichoptera, and Hynes (1955) for Plecoptera.

Data loggers (HOBO**®** pendant Temperature/Light 64Kdata logger, Onset Computer Corporations) were placed in the fissures in the autumn of 2014 which logged temperature every hour for approximately 1 year. Loggers were placed at 1, 5, and 10 m depth on one transect within each fissure for a depth gradient. Another two loggers were placed at 1 m depth on two other transects for horizontal comparison. Substrate type was noted at each sampling site. Chlorophyll *a* samples were collected at 2 and 4 m on three transects in each fissure in June 2015 and stored in black plastic bags. The 2 L samples were filtered through a 47 mm glass microfiber filter (GE Healthcare Life Sciences) and stored in 5 ml ethanol in a cooler for 24 hr. Subsequently, the samples were put in a centrifuge (Hettich Rotanta type 3500) at 1,660 *g* for 5 min and put into a 10 × 10 mm cuvette, and the absorbance was measured in a spectrophotometer (Hach DR 5000) at 665 and 700 nm. This was repeated after acidification with 0.1 N HCL which was used to convert Chl*a* to phaeophytin. Finally, conductivity, total dissolved solids, and pH were measured with a handheld instrument (YSI Pro 1030 pH/ORP/Conductivity/Temperature Instrument, YSI Incorporated) in September 2015.

Average densities (#/m^2^) were calculated for all species at all sampling sites within each fissure (Appendix 1). Shannon diversity and mean densities of common taxa were compared among fissures using Wilcoxon test. Communities within fissures were compared between rock wall and bottom using Wilcoxon test, and between different depths on bottom using Spearman rho test. Principal component analysis (PCA) was performed to compare macroinvertebrate community structure between fissures. Finally, community structure was again analyzed in relationship to substrate type (rock wall vs. bottom) and depth using redundancy analysis (RDA). For all statistical tests, significance level was set at 0.05. All statistical analyses were performed using RStudio version 0.98.1062 (R Core Team, 2013), package Vegan (Oksanen et al., 2013) and Canoco Version 4.5.

## 3 RESULTS

**Figure 4:**
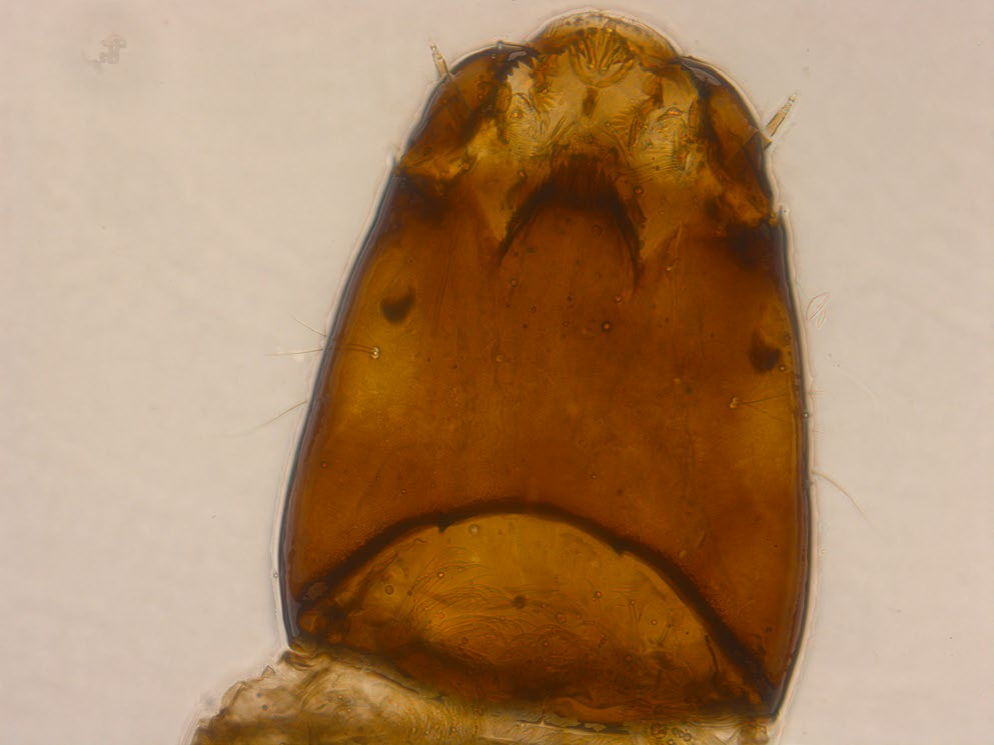
The most common species found in fissures Flosagjá and Silfra, the chironomid *Diamesa zernyi*

A total of 32 invertebrate taxa were found during the study (Table 1). Taxa richness was 24 and 25 in Flosagjá and Silfra, respectively. Of these taxa, 18 were shared between both fissures. The most common species was the chironomid *Diamesa zernyi* (Figure 4). Small benthic Arctic charr was also observed in all three fissures.

**Table 1:** List of species of Chironomidae, Cladocera, Copepoda, Ostracoda, Plecoptera, Trichoptera, and other taxa groups collected and identified in two cold groundwater fissures in Iceland (Flosagjá and Silfra)

	Flosagjá	Silfra
Chironomidae		
*Chaetocladius vitellinus* gr. (Kieffer, 1908)		+
*Cricotopus tibialis* gr. (Meigen, 1804)	+	
*Diamesa bertrami* (Edwards, 1935)		+
*Diamesa zernyi* gr. (Edwards, 1933)	+	+
*Eukiefferiella minor* (Edwards, 1929)	+	+
*Macropelopia* sp.	+	
*Micropsectra* sp.	+	+
*Orthocladius frigidus* (Zetterstedt, 1838)	+	+
*Orthocladius oblidens* (Walker, 1856)	+	+
*Rheocricotopus* cf*. effusus* (Walker, 1856)	+	+
*Thienemaniella* sp.	+	+
Cladocera		
*Acroperus harpae* (Baird, 1834)	+	+
*Alona affinis* (Leydig, 1860)	+	+
*Alona quadrangularis (*Müller, 1785)	+	+
*Alona werestschagini* (Sinev, 1999)	+	
*Chydorus* cf*. sphaericus (*Müller, 1776)	+	+
*Macrothrix hirsuticornis *(Norman and Brady, 1867)	+	+
Copepoda		
*Acanthocyclops robustus* (Fischer, 1853)		+
*Bryocamptus zschokkei* (Schmeil, 1823)		+
*Diacyclops bisetosus* (Rehberg, 1880)	+	
*Eucyclops* sp.		+
*Megacyclops viridis* (Jurine, 1820)	+	+
Ostracoda		
*Cypridoidea* sp.	+	+
*Fabaeformiscandona* sp.	+	+
*Potamocypris zschokkei* (Kaufmann, 1900)	+	
Plecoptera		
*Capnia vidua* (Klapálek, 1904)		+
Trichoptera		
*Apatania zonella* (Zetterstedt, 1840)	+	+
Other taxa		
Acarina	+	+
Collembola	+	
Empididae		+
*Salvelinus alpinus* (Linnaeus, 1758)	+	+
Total no. taxa	24	25

**Figure 5:**
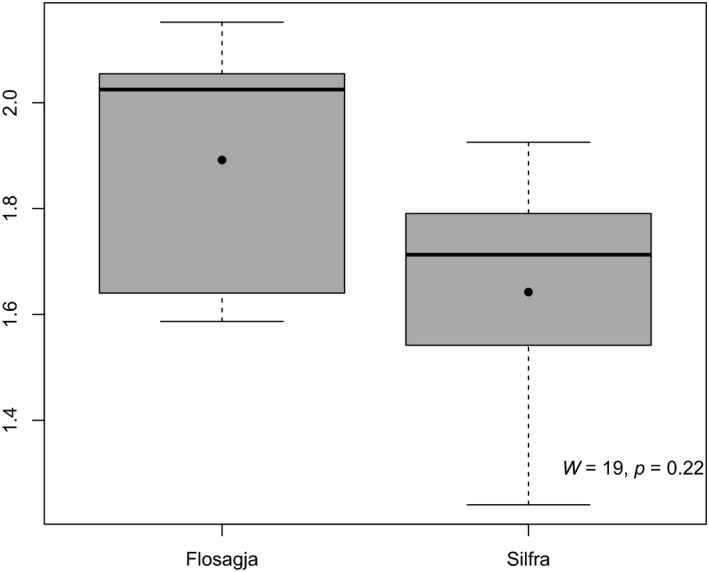
Shannon diversity for Flosagjá and Silfra with range of values calculated from all sampling stations, mean (black dot) and median (black line). Whiskers reflect variability beyond the upper and lower quartile; the 3rd (Q3) and 1st (Q1) quartiles are demarcated by top and bottom of boxes. Empty circles are outliers. Result from a Wilcoxon signed‐rank test (*p* < 0.05) comparing Shannon diversity index between fissures is found in the lower right corner

**Figure 6:**
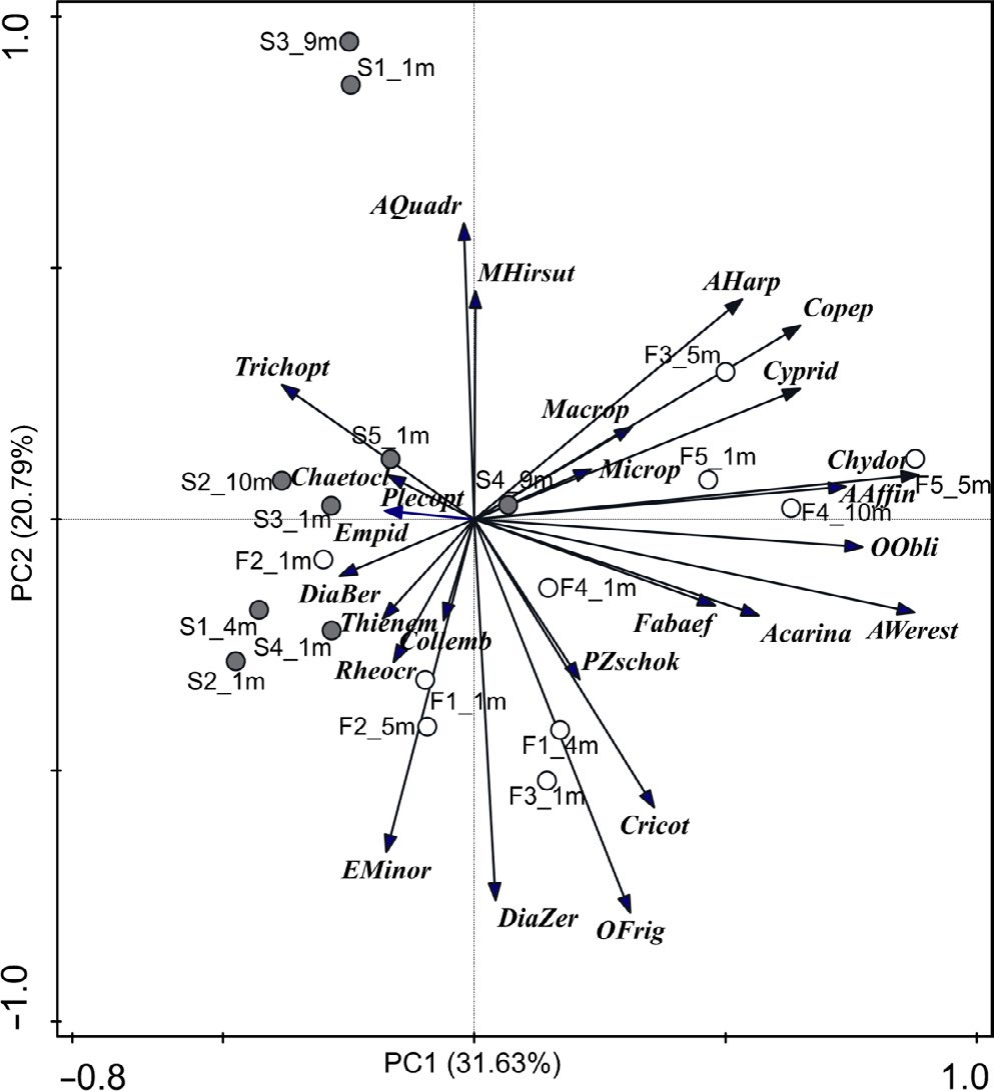
PCA ordination diagram of all taxa (in italics) and sampling sites within Flosagjá (white circles) and Silfra (gray circles)

Shannon diversity was 2.1 in Flosagjá fissure and 1.7 in Silfra, and there was no significant difference in Shannon diversity between fissures (Figure 5). Macroinvertebrate assemblages were similar except for cladocerans, which were found in greater densities in Flosagjá compared to Silfra (W = 87, *p* = 0.005, Wilcoxon signed‐rank test). This difference is mostly attributed to *Chydorus* sp. which was the most common cladoceran, while rare in Silfra. *Alona werestschagini*, which was recently documented in Iceland for the first time (Novichkova, Chertoprud, & Gíslason, 2014), was also entirely absent from Silfra while being the second most common cladoceran in Flosagjá. Copepods were treated as one taxonomic group in analysis as only a subsample was identified to the species level. Copepods were found in similar densities in the two fissures (*W* = 58.5, *p* = 0.54, Wilcoxon signed‐rank test) but species assemblages varied and fewer copepod species were identified in Flosagjá (Table 1). Chironomid and Ostracod assemblages were similar between the two fissures and found in similar densities (Chironomidae: *W* = 57, *p* = 0.63; Ostracoda: *W* = 66, *p* = 0.24, Wilcoxon signed‐rank test). Although Tricoptera and Plecoptera were more common in Silfra, there was no significant difference in densities between the two fissures (Trichoptera: *W* = 13.5, *p* = 0.12; Plecoptera: *W* = 10, *p* = 0.45). A PCA ordination revealed that taxa composition between the fissures is distinct although assemblages overlap to some degree (Figure 6). Crustacean taxa were more common and found in higher densities in Flosagjá fissure while aquatic insect larvae were more abundant in Silfra. Altogether, 31.6% of the variance in taxa composition could be explained by a gradient along the first PCA axis, while 20.8% of the variance could be explained by a gradient along the second axis.

**Figure 7:**
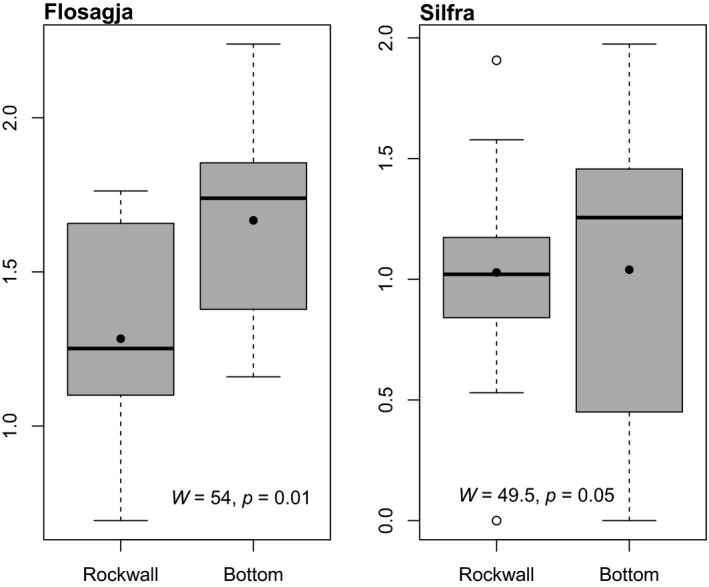
Shannon diversity index for invertebrate communities on different substrate types (Rock wall and bottom) in Flosagjá and Silfra with mean (black dot) and median (black line). Whiskers show variability beyond the upper and lower quartile; the 3rd (Q3) and 1st (Q1) quartiles are demarcated by top and bottom of boxes. Empty circles are outliers. Results from Wilcoxon signed‐rank test (*p* < 0.05) comparing Shannon diversity index between substrate types are shown for each fissure

Samples were taken on the two substrate types found in fissures, rock walls and bottom. While there was no significant difference in Shannon diversity between rock wall and bottom in Silfra, there was a significant difference in Shannon diversity between substrate types in Flosagjá (Figure 7). For most taxonomic groups, there was no significant difference found in densities on rock wall versus bottom in either fissure. The only exception was ostracods in Flosagjá, which were found in significantly higher densities on bottom (*W* = 1, *p* = 0.02, Wilcoxon rank sum test).

**Figure 8:**
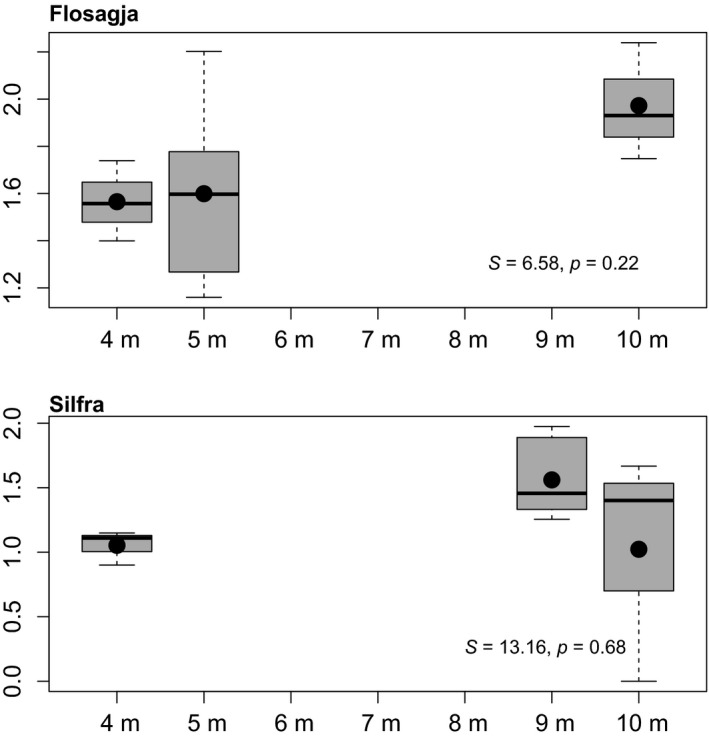
Shannon diversity index for invertebrate communities at different depth on bottom in Flosagjá and Silfra with mean (black dot) and median (black line). Whiskers show variability beyond the upper and lower quartile; the 3rd (Q3) and 1st (Q1) quartiles are demarcated by top and bottom of boxes. Empty circles are outliers. Results from Spearman rho test (*p* < 0.05) comparing Shannon diversity index between depths are shown in the bottom right corner for each fissure

**Figure 9:**
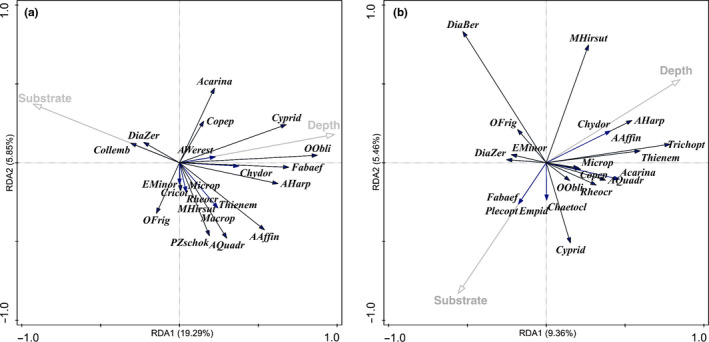
(a‐b) RDA ordination diagram of taxa (in italics with black arrows) and environmental variables (Substrate and Depth with gray arrows) in Flosagjá (a) and Silfra (b)

Finally, there was no significant difference in Shannon diversity on the bottom at different depths in either fissure (Figure 8) nor was there a significant difference in invertebrate density at different depths in fissures (Flosagjá: *S* = 0.22, *p* = 0.72; Silfra: *S* = −0.32, *p* = 0.68, Spearman rank correlation test). An RDA analysis was also performed on assemblages in response to depth and substrate type (rock wall vs. bottom) within fissures. Neither variable significantly explained species composition in either Flosagjá or Silfra (Figure 9a and 9b).

Water temperature in fissures was low with an average of 3.6°C in Flosagjá and 3.5 in Silfra and low chl*a* concentrations at an average of 0.01 in Flosagjá and 0.04 in Silfra (Table 2) making both fissures oligotrophic (Carlson & Simpson, 1996). No zooplankton was observed in water samples collected for chl*a* measurements. The conductivity, pH, and total dissolved solids in the fissures were not significantly different. Detailed identification of flora in fissures was not conducted but a subsample of biofilm collected along invertebrate samples from both fissures was examined. The biofilm was mostly composed of Cyanobacteria and benthic diatoms. The green algae *Tetraspora cylindrica* and *Klebsormidium* sp. were also observed in Silfra fissure.

**Table 2:** Mean annual temperature, one measurement made per hour (September 2014–September 2015) with standard deviation (*SD*), minimum values (Min), and maximum values (Max), chl*a* concentrations (June 2015) with *SD*, Min, and Max values as well as pH, conductivity, and total dissolved solids (TDS) (September 2015)

Fissure	Temperature (°C)				Chlorophyll *a* (µg L^−1^)				pH	Conductivity (µS cm^−1^)	TDS (mg L^−1^)
	Mean	*SD*	Min	Max	Mean	*SD*	Min	Max			
Flosagjá	3.61	1.05	3.26	4.62	0.01	0.06	−0.08	0.08	9.8	83.2	54.0
Silfra	3.53	0.6	3.37	4.10	0.04	0.05	0.00	0.15	9.5	70.5	45.2

## 4 DISCUSSION

The main aim of this study was to investigate invertebrate biodiversity in groundwater fissures and compare assemblages between fissures with different morphological characteristics. A second main aim of this study was to determine whether different substrate types and variable depth in fissures were influencing species diversity and distribution of organisms. Shannon diversity was higher in Flosagjá but no significant difference was found in Shannon diversity between fissures. Assemblages did, however, vary to some degree, and cladocerans were found in higher densities in Flosagjá. The fissure Silfra opens into Lake Þingvallavatn (Malmquist, 2012) and has a more rapid water current compared to the landlocked Flosagjá, where a slower water current is likely to create a more suitable habitat for smaller crustaceans such as cladocerans. In a study investigating how environmental variables shape invertebrate communities in Icelandic springs, Govoni et al. (2018) found a similar pattern where crustaceans and in particular cladocerans were most common in limnocrene springs. Meanwhile, *Diamesa* spp. were most abundant in rheocrene springs. Although water current is more pronounced in Silfra compared to Flosagjá, Silfra cannot be considered a true rheocrene spring (Springer & Stevens, 2008). Chironomids including *D. zernyi* were more common in Silfra but there was no significant difference in chironomid density between the two fissures and all taxa groups besides crustaceans were found in similar densities. The taxa composition in the fissures is comparable to other freshwater habitats in Iceland, with chironomids being the dominant invertebrate group (Gíslason et al., 1998).

One might expect the distribution of taxa within a fissure to be influenced by variation in depth and the different types of substrate (rock wall and bottom), which could create dissimilar habitats with unique characteristics and influence the distribution of resources. There was a difference in Shannon diversity between substrate types in Flosagjá fissure, and this was attributed primarily to higher densities of ostracods found at the bottom. The distribution of all other taxa groups was relatively even within fissures. The biofilm mats that cover the entire substrate in fissures may help to explain this homogeneity and obscure the effects of the morphological differences. While water samples had very low concentrations of chl*a* and contained no zooplankton, these wall/benthic mats seem to create favorable habitat for invertebrates and are akin to those commonly found in high latitude freshwater systems. In systems where the water temperature is below 5°C, the primary production of phytoplankton has been shown to be nutrient limited, while benthic cyanobacterial mats on the contrary act as a type of microenvironment that is nutrient sufficient, rich in biomass, and is home to many species (Bonilla, 2005). Detailed identification of flora in fissures is still lacking but based on subsamples that were analyzed during the project the biofilm mats appear to be mostly consist of Cyanobacteria and benthic diatoms. Mat‐forming diatoms and Cyanobacteria have been documented in cold freshwater systems in the Arctic and Antarctic (Ohtsuka, Kudoh, Imura, & Ohtani, 2006; Rautio & Vincent, 2007), and many studies have identified such biofilm as an important carbon source for invertebrates (Hall & Meyer, 1998; Hansson & Travik, 2003; Rautio & Vincent, 2007). The Cyanobacteria genus *Oscillatoria* forms algal mats, which are known to grow around artesian springs at the bottom of Lake Þingvallavatn where nitrogen‐rich water is discharged. As long as the nitrogen availability is sufficient, Cyanobacteria of this genus can photosynthesize under very low light conditions (Jónsson, Gunnarsson, & Jónasson, 2011). Cyanobacteria seem to cope well within caverns and in narrow fissures where light penetration may be limited. Since such biofilm mats cover the entire substrate in fissures, they may continue to provide suitable habitat and be a food source for invertebrates regardless of substrate type.

During the project, a new fissure was discovered in Þingvellir National park and was named Huldugjá (The Hidden fissure). This fissure is underground and is approximately 80 m long and 2 m wide and reaches a depth of 40 m making it among the deepest accessible fissures in Iceland. Five samples for qualitative analysis were collected in Huldugjá fissure, and two species were found: Copepod *Megacyclops viridis*, which was also common in the open water fissures, and a groundwater amphipod *Crangonyx islandicus* that is endemic to Iceland (Svavarsson & Kristjánsson, 2006). *C. islandicus* was seen moving around and looked healthy, while the *M. viridis* specimen appeared less healthy and had indications of deterioration of the tissues. This specimen may have been swept into the cave although there is also a slight possibility that sampling equipment was contaminated from earlier sampling procedures. As this is so far the only known fissure in Iceland that is completely underground, it may prove to be an interesting study site for future research on groundwater ecosystems and cave populations.

Benthic invertebrates provide essential ecosystem services by transferring energy through trophic levels and by mixing sediments (Covich et al., 1999). Furthermore, invertebrates play a key role in nutrient cycling along with fungi and bacteria (Vanni, 2002). Scientists are still working on unraveling the complex connections within ecosystems and the role of organisms and biodiversity in their functioning. Meanwhile, there are signs that we are losing many species before we ever get a chance to study them (Covich et al., 1999; Singh, 2002). Far too often, freshwater invertebrate assemblages are overlooked and their importance for ecosystem services is not realized until unforeseen changes occur (Covich et al., 1999). Invertebrates make up most of the fauna in groundwater fissures in Iceland.

## 5 CONCLUSIONS

Fissures that form on a divergent plate boundary can be accessed in very few places in the world as these are ordinarily suboceanic features. In Iceland, they are subaerial and clearly visible in many locations. Often they create an opening into the groundwater aquifer and facilitate spring flow in what are some of the largest cold water spring systems in the world (Óskarsdóttir, 2011; Sigurðsson & Sigbjarnason, 2011). Some groundwater fissures have become popular tourist destinations in recent years. Silfra fissure in particular has experienced a rapid increase in visitors for the past decade and is under considerable anthropogenic pressure. Currently, however there is no monitoring of the biological communities in fissures and their ecosystems are poorly known. Here we provided a detailed first look at benthic macroinvertebrate communities in two oligotrophic groundwater fissures in Iceland in relationship to morphological habitat. The fissures studied have cold oligotrophic water and high pH and are inhabited by a diversity of benthic macroinvertebrates. Invertebrate assemblages were similar between fissures with different morphological characteristics, and the taxa identified are evenly distributed on substrate despite the heterogenous habitat. The uniform distribution of macrozoobenthos in fissures may be the result of benthic biofilm mats providing suitable microhabitat for invertebrates regardless of depth and type of substrate. Biofilm mats in cold nutrient‐limited systems have been shown to be an important carbon source and habitat for invertebrates (Bonilla, 2005; Hall & Meyer, 1998; Hansson & Travik, 2003; Rautio & Vincent, 2007). Therefore, the health of invertebrate communities in fissures is likely to be related to the health of the biofilm mats.

## CONFLICT OF INTEREST

The authors declare no conflicts of interests.

## AUTHOR CONTRIBUTIONS

J.H.Ó., J.G.Þ., B.K.K., and J.S.Ó. designed the project. J.H.Ó. and J.G.Þ. carried out the sampling and taxa identifications. J.H.Ó. lead the writing of the manuscript. All authors discussed the results and contributed to the final manuscript.

## Data Availability

The original data including the sampling locations and density of invertebrate taxa at each sampling station have been submitted to the Dryad data repository, https://doi.org/10.5061/dryad.rr2vp6g.

## APPENDIX 1

### MEAN DENSITIES OF INVERTEBRATES (M^2^) AT DIFFERENT DEPTHS IN FLOSAGJÁ WITH STANDARD DEVIATION (*SD*)

**Table:**

1 m		4 m		5 m		10 m	
Mean	*SD*	Mean	*SD*	Mean	*SD*	Mean	*SD*
Chironomidae								
*Chaetocladius vitellinus group*	0.0	0.0	0.0	0.0	0.0	0.0	0.0	0.0
*Cricotopus tibialis group*	45.0	67.6	150.0	132.3	61.1	71.9	24.0	34.5
*Diamesa bertrami*	0.0	0.0	0.0	0.0	0.0	0.0	0.0	0.0
*Diamesa zernyi group*	335.0	326.9	258.3	101.0	144.4	145.1	48.4	78.4
*Eukiefferiella minor*	60.0	79.5	125.0	75.0	27.8	47.0	15.7	15.0
*Macropelopia *sp.	0.0	0.0	0.0	0.0	2.7	8.3	0.0	0.0
*Micropsectra* sp.	1.7	6.5	0.0	0.0	5.6	16.7	5.6	3.2
*Orthocladius frigidus*	325.0	225.6	641.7	316.0	166.7	227.1	75.7	91.8
*Orthocladius oblidens*	5.0	14.0	33.3	57.7	19.4	31.7	10.6	11.1
*Rheocricotopus cf. effusus*	1.7	6.5	0.0	0.0	5.6	16.7	5.6	3.2
*Thienemaniella *sp.	0.0	0.0	0.0	0.0	5.6	16.7	5.6	3.2
Cladocera								
*Acroperus harpae*	1.7	6.5	16.7	28.9	2.8	91.0	30.3	1.6
*Alona affinis*	1.7	6.5	16.7	28.9	19.4	60.1	20.0	11.2
*Alona quadrangularis*	0.0	0.0	8.3	14.4	11.1	33.3	0.0	0.0
*Alona werestschagini*	50.0	114.2	41.7	14.4	80.6	166.8	55.6	45.4
*Chydorus cf. sphaericus*	33.3	57.2	8.3	14.4	200.0	379.8	126.6	114.8
*Macrothrix hirsuticornis*	0.0	0.0	0.0	0.0	11.1	33.3	0.0	0.0
Ostracoda								
*Cypridoidea* sp.	16.7	29.4	16.7	14.4	58.3	104.2	34.7	33.4
*Fabaeformiscandona* sp.	0.0	0.0	8.3	14.4	2.8	8.3	2.8	1.6
*Potamocypris zschokkei*	3.3	8.8	8.3	14.4	16.7	41.5	13.8	9.6
Plecoptera								
*Capnia vidua*	0.0	0.0	0.0	0.0	0.0	0.0	0.0	0.0
Trichoptera								
*Apatania zonella*	1.6	6.5	0.0	0.0	0.0	0.0	0.0	0.0
Other groups								
Acarina	23.3	29.1	0.0	0.0	19.4	38.4	12.8	10.9
Collembola	1.7	6.5	0.0	0.0	0.0	0.0	0.0	0.0
Copepoda	75.0	168.5	8.3	14.4	27.8	80.0	26.7	14.3
Empididae	0.0	0.0	0.0	0.0	0.0	0.0	0.0	0.0
Gastropoda	15.0	13.3	0.0	0.0	0.0	0.0	0.0	6.7
0.0	0.0	0.0	0.0	0.0	0.0	0.0	0.0

### Mean densities of invertebrates (m^2^) at different depths in Silfra with standard deviation (*SD*)

**Table:**

1 m		4 m		9 m		10 m	
Mean	*SD*	Mean	*SD*	Mean	*SD*	Mean	*SD*
Chironomidae								
*Chaetocladius vitellinus group*	15.0	13.3	0.0	0.0	0.0	0.0	4.1	14.4
*Cricotopus tibialis group*	0.0	0.0	0.0	0.0	0.0	0.0	0.0	0.0
*Diamesa bertrami*	0.0	0.0	8.3	14.4	0.0	0.0	0.0	0.0
*Diamesa zernyi group*	483.3	440.7	350.0	264.6	245.8	280.4	46.7	89.3
*Eukiefferiella minor*	190.0	175.3	216.7	203.6	208.3	257.7	43.0	17.9
*Macropelopia *sp.	0.0	0.0	0.0	0.0	0.0	0.0	0.0	0.0
*Micropsectra *sp.	6.7	15.3	0.0	0.0	33.3	54.0	9.0	15.9
*Orthocladius frigidus*	91.7	65.4	225.0	50.0	120.8	160.0	26.7	79.2
*Orthocladius oblidens*	5.0	10.6	0.0	0.0	12.5	30.6	5.1	6.7
*Rheocricotopus cf. effusus*	1.7	6.7	0.0	0.0	0.0	0.0	4.2	14.4
*Thienemaniella *sp.	0.0	0.0	0.0	0.0	0.0	0.0	4.2	14.4
Cladocera								
*Acroperus harpae*	0.0	0.0	0.0	0.0	33.3	28.9	0.0	0.0
*Alona affinis*	0.0	0.0	0.0	0.0	29.2	48.5	8.1	14.6
*Alona quadrangularis*	6.7	11.7	0.0	0.0	25.0	31.6	5.3	11.9
*Alona werestschagini*	0.0	0.0	0.0	0.0	0.0	0.0	0.0	0.0
*Chydorus cf. sphaericus*	0.0	0.0	0.0	0.0	8.3	20.4	3.4	4.2
*Macrothrix hirsuticornis*	0.0	0.0	16.7	14.4	25.0	38.7	6.5	10.4
Ostracoda								
*Cypridoidea *sp.	45.0	87.4	0.0	0.0	37.5	68.5	11.4	41.4
*Fabaeformiscandona* sp.	1.7	6.7	0.0	0.0	0.0	0.0	0.0	3.3
*Potamocypris zschokkei*	0.0	0.0	0.0	0.0	0.0	0.0	0.0	0.0
Plecoptera								
*Capnia vidua*	1.7	6.7	0.0	0.0	0.0	0.0	0.0	0.0
Trichoptera								
*Apatania zonella*	5.0	16.9	0.0	0.0	20.8	33.2	5.5	2.8
Other groups								
Acarina	10.0	23.4	0.0	0.0	33.3	43.8	7.3	16.9
Collembola	0.0	0.0	0.0	0.0	0.0	0.0	0.0	0.0
Copepoda	38.3	29.2	8.3	14.4	95.8	103.0	17.2	40.2
Empididae	1.7	6.7	0.0	0.0	0.0	0.0	0.0	0.0
Gastropoda	15.0	13.3	0.0	0.0	0.0	0.0	0.0	0.0
